# MRI of giant cell tumour of larynx: marked *T*_2_ hypointensity due to abundant haemosiderin deposition

**DOI:** 10.1259/bjrcr.20150388

**Published:** 2016-05-24

**Authors:** Etsushi Iida, Matakazu Furukawa, Naofumi Matsunaga, Yoshimi Anzai

**Affiliations:** ^1^Department of Radiology, Yamaguchi University, Graduate School of Medicine, Yamaguchi, Japan; ^2^Department of Radiology, University of Utah Health Sciences Center, Salt Lake City, UT, USA

## Abstract

Giant cell tumours (GCTs) are benign tumours commonly found in the long bones. Rarely, they may occur in the larynx, often resulting in hoarseness and anterior neck swelling. Since Wessely reported the first case of laryngeal GCT in 1940, 35 cases have been identified. Herein, we present a case of a 53-year-old male with GCT of the larynx that showed *T*_1_ and *T*_2_ hypointense signal on MRI, presumably owing to abundant haemosiderin deposition. We also discuss the imaging findings of CT and ^18^F-fludeoxyglucose positron emission tomography scans, as well as the pathological correlation.

## Summary

Giant cell tumours (GCTs) are benign tumours commonly found in the long bones. Rarely, they may occur in the larynx, often resulting in hoarseness and anterior neck swelling. Since Wessely^1^ reported the first case of laryngeal GCT in 1940, 35 cases have been identified. Herein, we present a case of a 53-year-old male with GCT of the larynx that showed *T*_1_ and *T*_2_ hypointense signal on MRI, presumably owing to abundant haemosiderin deposition. We also discuss the imaging findings of CT scan and ^18^F-fludeoxyglucose positron emission tomography (FDG-PET) scans, as well as the pathological correlation.

## Clinical presentation

A 53-year-old male complained of hoarseness and presented at the ENT clinic. Laryngoscopic examination revealed polypoid degeneration of the right true vocal cord. In the course of the clinical observation of the polypoid degeneration of the true vocal cord for 2 years, a submucosal swelling of the false vocal cord (FVC) was found on laryngoscopic examination. CT scan, MRI and FDG-PET scan were performed as imaging work-ups of the FVC submucosal lesion. The patient had normal renal function.

## Imaging findings

**Figure 1.**
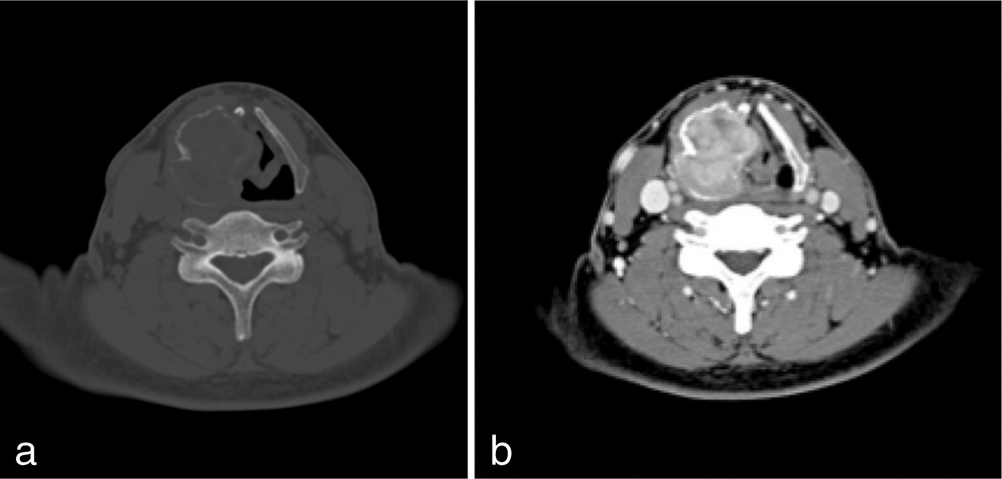
(a) Axial unenhanced CT scan (bone window) showing an expansile mass in the right plate of the thyroid cartilage with extensive erosion and thinning of the cortex. (b) Axial enhanced CT scan (soft tissue window) revealing heterogeneous enhancement in the tumour.

Unenhanced CT scan revealed an expansile heterogeneously iso-low attenuated mass centred in the right thyroid cartilage, measuring 40 × 45 × 46 mm in size. The right thyroid cartilage was entirely replaced by the tumour, associated with erosion of the cortex (Figure 1a). On enhanced CT scan, heterogeneous enhancement was seen in the tumour (Figure 1b). The lesion was submucosal and displaced the laryngeal mucosa medially at the FVC level.

**Figure 2.**
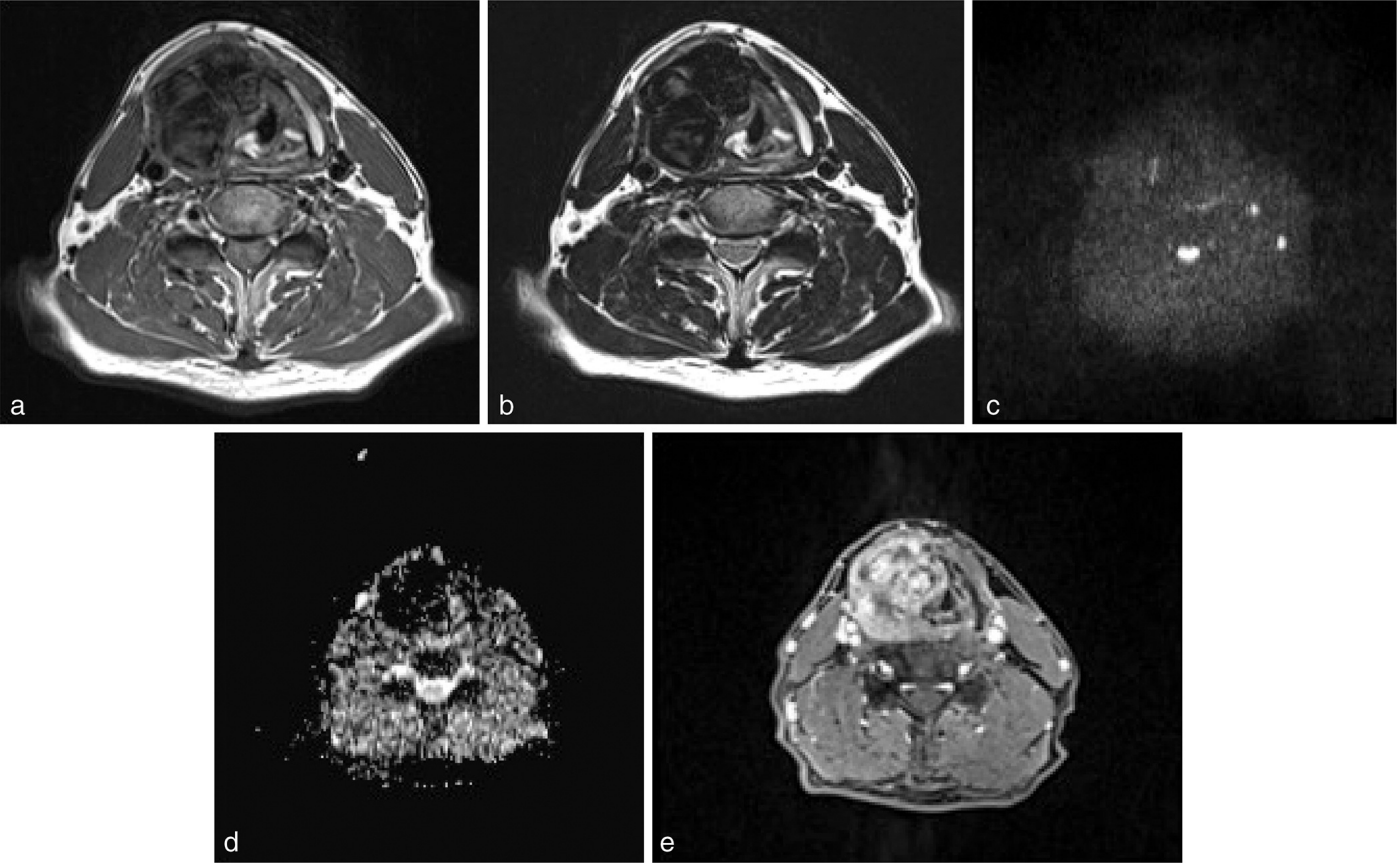
MRI of the larynx. Most of the tumour showed marked hypointensity on both *T*_1_ (a; FSE, TR/TE = 584/12) and *T*_2_ weighted images (b; FSE, TR/TE = 4760/82). Diffusion-weighted image (c; spin-echo single-shot echo-planar sequence, TR/TE = 15,000/80, *b* factors of 0 and 1000 s mm ^−2^) and apparent diffusion coefficient maps (d) also showed hypointensity owing to the susceptibility effect. The tumour showed heterogeneous enhancement on post-contrast *T*_1_ weighted image with fat suppression (e; GRE, TR/TE = 3.78/1.4). The left side of the thyroid cartilage was not involved in the tumour. FSE, fast spin echo; GRE, gradient echo sequence; TE, echo time; TR, repetition time.

On 3 T MRI, most of the tumour showed marked hypointensity on both *T*_1_ and *T*_2_ weighted images (Figure 2a,b). Diffusion-weighted images showed lack of restricted diffusion (Figure 2c). Hypointensity on apparent diffusion coefficient maps was likely owing to the susceptibility effect from a haemorrhage (Figure 2d). On post-contrast *T*_1_ weighted images, the tumour showed heterogeneous enhancement (Figure 2e).

**Figure 3.**
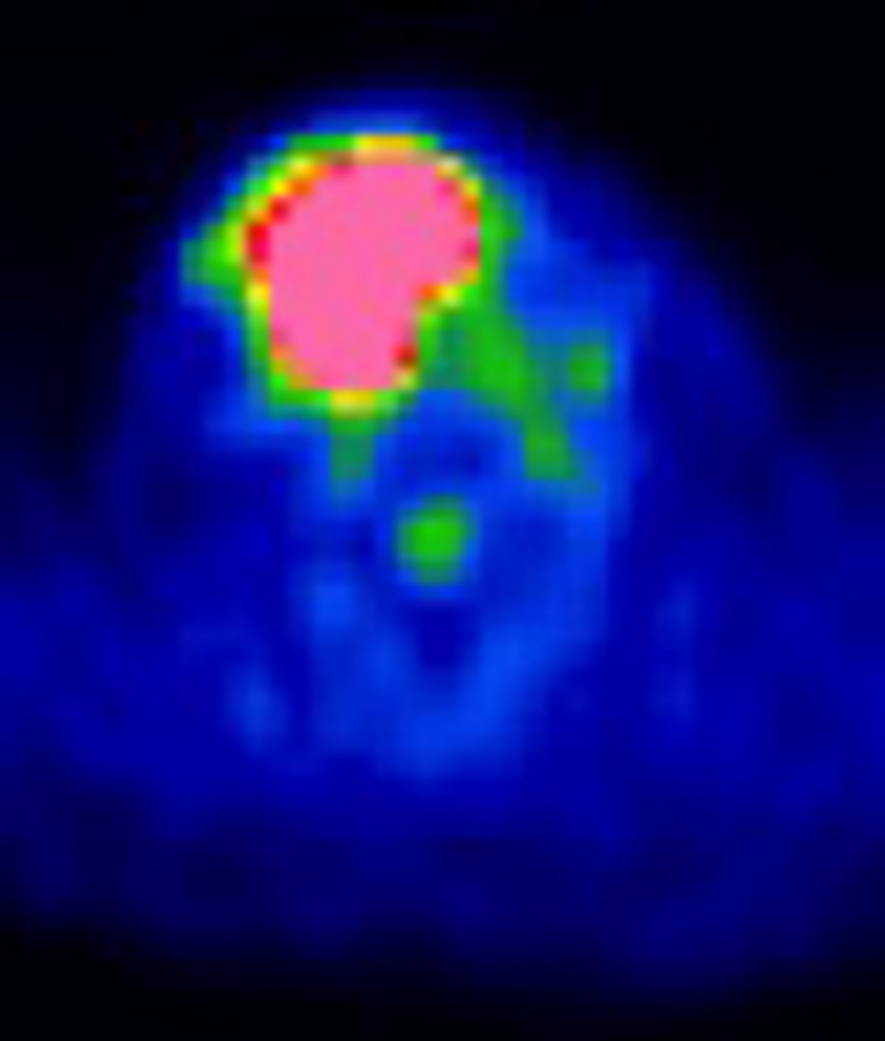
Abnormal high uptake (maximum standardized uptake value of 12.3) was seen in the tumour on ^18^F-fludeoxyglucose positron emission tomography imaging.

FDG-PET revealed abnormal high uptake [maximum standardized uptake value (SUV) of 12.3] in the tumour (Figure 3). There was no finding that suggested lymph node metastases either on CT scan, MRI or FDG-PET scan.

## Treatment

Total laryngectomy was performed because a malignant tumour was suggested on open biopsy.

## Outcome

**Figure 4.**
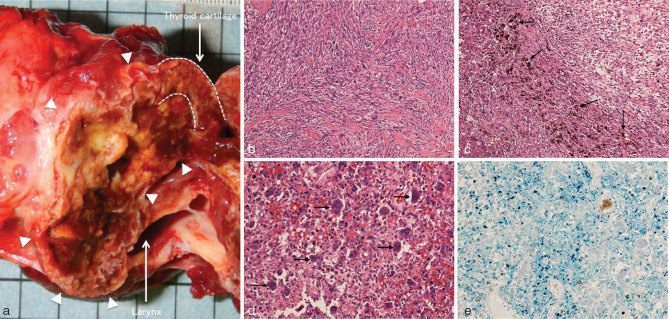
Histopathological images. (a) A photograph of the gross specimen. The resected specimen shows heterogeneous nature, including haemorrhage and degeneration in the tumour (arrowheads). (b) A proliferation of spindle-shaped cells arranged in fascicular or vague storiform pattern (haematoxylin and eosin, original magnification ×100). (c, d) Haemosiderin deposition (arrows in c) and osteoclast-like giant cells (arrows in d) were seen in the tumour (haematoxylin and eosin, original magnification ×200). (e) Most of the tumour was stained with Prussian blue, which suggested iron deposition (Prussian blue stain, original magnification ×200).

On gross examination, the resected specimen had a well-defined lobulated shape with heterogeneous nature, including haemorrhage and necrotic degeneration (Figure 4a). On microscopic examination, a proliferation of spindle-shaped cells in a fascicular or vague storiform pattern (Figure 4b) accompanied by osteoid formation with focal osteoblastic rimming, haemosiderin deposition (Figure 4c) with variable stages of haemorrhage and aggregates of osteoclast-like giant cells (Figure 4d) were seen. Most of the tumour was strongly stained with Prussian blue (Figure 4e), which suggested abundant iron deposition owing to haemorrhage. Pleomorphic tumour cells with hyperchromatic nuclei and occasional mitotic figures were seen. Both giant and spindle cells were positive for a mesenchymal marker, vimentin and a macrophage marker, cluster of differentiation 68, on immunohistochemical staining. Spindle cells were also positive for α-smooth muscle actin. However, both giant and spindle cells were negative for epithelial membrane antigen , cytokeratin, desmin, anaplastic lymphoma kinase, CD34, CD31 and human melanin black 45. There was no evidence of high-grade malignancy. The final pathological diagnosis was that of a GCT arising from the thyroid cartilage. The patient is currently free of recurrence 2.5 years after the laryngectomy.

## Discussion

GCTs account for about 4–9.5% of all primary bone tumours and usually show a slight female predilection, with ratios ranging from 1.1 : 1 to 1.5 : 1, and often occur in the third decade of life.^2,3^ The most common site is the distal femur (23–30% of cases), followed by the proximal tibia (20–25%), distal radius (10–12%) and sacrum (4–9%).^^2^^

GCT of the head and neck region is rare, and accounts for approximately 2% of all GCTs.^3^ The majority of head and neck GCTs occur at the skull base and in the temporal bones.^3^ Primary giant cell tumours of the larynx (GCTL) are exceedingly rare, show male predilection and often occur in patients aged between 23 and 67 years (average age, 42.7 years).^^4^^ The most common site is the thyroid cartilage, followed in order by the cricoid and the epiglottic cartilage.^3,5–7^ Clinical symptoms of GCTL are hoarseness, dyspnoea, dysphagia, pain and palpable neck mass.^3,5,7^ The tumours are usually submucosal and protrude into the endolarynx. Endoscopic examination might show submucosal swelling of the vocal cord and/or the FVC, but the laryngeal mucosa is usually intact and does not show an ulcerative or haemorrhagic change unlike squamous cell carcinoma.^3,5^

### Imaging findings of GCTL

On CT scan, GCTL shows a homogeneous soft tissue mass with cortical expansion and destruction of the thyroid cartilage.^3,5^ The mass might extend into the soft tissues of the larynx and into the paraglottic spaces.^6,7^ Small foci of calcifications and mild contrast enhancement within the tumour have been reported.^4,6,7^ In the present case, the tumour showed heterogeneous iso- to low density with heterogeneous contrast enhancement.

There are few reports of MRI findings for GCTL. The tumour showed hypointensity on *T*_1_ weighted images and a heterogeneous hyperintensity or hypo- to intermediate intensity on *T*_2_ weighted images.^3,5^ Heterogeneous enhancement on gadolinium-enhanced *T*_1_ weighted images is attributable to the histopathological findings with rich stroma containing numerous, thin-walled capillaries and secondary degeneration resulting in cyst formation.^5,7^

Chang et al^8^ reported that GCTL showed high uptake on FDG-PET (maximum SUV of 11.1), which was similar to the present case (maximum SUV of 12.3). Avid uptake of FDG in GCT is caused by an enhanced vascular fraction with increased ^18^F-FDG transport and overexpression of hexokinase-2, a key enzyme in the glycolytic pathway in both giant and spindle cells in GCT.^9,10^

In the present case, GCTL showed marked hypointensity on both *T*_1_ and *T*_2_ weighted images, as well as diffusion-weighted images and apparent diffusion coefficient, which are common (63%) in GCTs, and support the diagnosis of GCTs.^11^ These hypointense signals were explained by the paramagnetic susceptibility effect of abundant haemosiderin deposition due to extravasated or phagocytized erythrocytes in the tumour.^11^

### Differential diagnosis of GCTL based on imaging findings

Regarding the differential diagnosis of *T*_2_ hypointense signal in the laryngeal skeleton, undifferentiated pleomorphic sarcoma, previously named malignant fibrous histiocytoma, should be considered. A certain histopathological subtype, formerly known as giant cell malignant fibrous histiocytoma, can contain abundant giant cells and haemosiderin deposition due to extravasated or phagocytized erythrocytes in the tumour.^6^

Giant cell reparative granuloma (GCRG) and brown tumour may also show hypointensity on *T*_2_ weighted images owing to their histological similarity to GCT.^7^ However, both GCRG and brown tumour are extremely rare in the larynx. GCRGs most frequently affect the mandible, maxilla, hands or feet, and brown tumours usually occur in patients with primary or secondary hyperparathyroidism, which was not seen in the present case.^2,4^

Other potential *T*_2_ hypointense laryngeal lesions include amyloidosis and sarcoidosis, which may present as laryngeal submucosal mass but not arise from the laryngeal cartilage.

Regarding the differential diagnosis of the tumour in the laryngeal skeleton, chondroma and chondrosarcoma should be included as frequent pathologies.^6^ These tumours usually show internal chondroid calcification on CT scan, hyperintensity on *T*_2_ weighted images and marked enhancement after administration of contrast.^12^ The other rare differential diagnoses include osteosarcoma, osteoblastoma and metastasis to the laryngeal skeleton.

Complete surgical resection is required for the treatment of GCT, and adjuvant therapy is unnecessary.^5,13^ Radiation therapy is currently not recommended because of the increased risk of sarcomatous change.^13^

## Conclusions

Primary GCT of the larynx is rare and should be considered as a differential diagnosis for *T*_2_ hypointense submucosal laryngeal mass.

## Learning points

1. GCTL can demonstrate MRI hypointense signal owing to susceptibility effect by haemosiderin deposition.
2. The differential diagnosis of GCTL should include GCRG and undifferentiated pleomorphic sarcoma, especially when susceptibility effect is seen in the lesion.
3. GCT of the larynx can present with high uptake on FDG-PET because of an enhanced vascular fraction with increased ^18^F-FDG transport.

## Consent

Written informed consent was obtained from the patient for publication of this case report and any accompanying images.
